# Cure for a Felon

**Published:** 1871-12

**Authors:** 


### CURE FOR A FELON.

If a he thief, hang him! if a sore finger, an exchange says it is a sure cure to put some coals into a pot, throw on these coals a handful each of hops, rye-flour, and brown sugar, and hold the affected part for fifteen minutes in the steam, and repeat it several times. A more certain cure is to bore a hole in the pot lid, stick the finger through, and let it stay there until the finger is burnt off, and that finger will never have a felon more! We warn our readers against paying attention to any newspaper paragraphs in reference to health and disease, unless the name of the writer is attached. There is a scientific cure for a felon always safe, always efficacious, and relieving instantaneously and always: get a physician to plunge his lancet down to the bone. A natural felon is a born thief; a physiological felon is a boil between the bone and the sinew, or "fascia" as doctors love to talk. When a boil is under the skin only, it is painful enough until it "breaks," that is until the skin divides or bursts and lets out the yellow matter; but when it is remembered that the sinew is as much tougher than the skin as a beef hide is tougher than paper, it is easy to see that the pain of a boil, under the sinew, is more terrible than one under the thin skin, and that it must take longer to make its way through the fascia than through the skin; hence, instead of passing many sleepless nights and agonizing days in waiting for the matter to be absorbed, or make its way through the tough tendon, the educated surgeon advises the use of the lancet as above; for the cure is just as certain, and the relief from the agonizing pain is just as instantaneous, as in the case of the extraction of an aching tooth. The cause of a felon is usually a bruise of the finger heavy enough to reach down to the bone and to inflame it.

---

The most lamented death in New York for many years was greatly hastened, if not directly caused, by allowing the fire to go entirely out during a very cold night, — the death of a lady writer, loved and admired by many thousands.

